# No Effect of Host Species on Phenoloxidase Activity in a Mycophagous Beetle

**DOI:** 10.1371/journal.pone.0141167

**Published:** 2015-10-29

**Authors:** Vincent Formica, Amanda Kar-Men Chan

**Affiliations:** Department of Biology, Swarthmore College, Swarthmore, Pennsylvania, United States of America; Uppsala University, SWEDEN

## Abstract

Ecological immunology is an interdisciplinary field that helps elucidate interactions between the environment and immune response. The host species individuals experience have profound effects on immune response in many species of insects. However, this conclusion comes from studies of herbivorous insects even though species of mycophagous insects also inhabit many different host species. The goal of this study was to determine if fungal host species as well as individual, sex, body size, and host patch predict one aspect of immune function, phenoloxidase activity (PO). We sampled a metapopulation of *Bolitotherus cornutus*, a mycophagous beetle in southwestern Virginia. *B*. *cornutus* live on three species of fungus that differ in nutritional quality, social environment, and density. A filter paper phenoloxidase assay was used to quantify phenoloxidase activity. Overall, PO activity was significantly repeatable among individuals (0.57) in adult *B*. *cornutus*. While there was significant variance among individuals in PO activity, there were surprisingly no significant differences in PO activity among subpopulations, beetles living on different host species, or between the sexes; there was also no effect of body size. Our results suggest that other factors such as age, genotype, disease prevalence, or natal environment may be generating variance among individuals in PO activity.

## Introduction

The goal of ecological immunology is to understand the evolutionary processes and ecological factors that affect immune function and infection in the wild [1, 2]. Diet, sex, body size, geographic location, social environment, and even micro-environmental differences among populations are all ecological factors shown to influence immune response in insects [3–8]. Host species can be a major source of environmental variation across several of these variables within and among insect populations. For instance, the species and quality of the host plants of herbivorous insect can affect several components of immune response [9–11]. Ecological factors that are influenced by the host, such as density of the population, nutritional quality, or risk of predation have also been shown to affect immune function in insects [12–14].

Emerging work on the effects of host species on immune response is largely centered on herbivorous insects [4, 7, 9, 15, 16]. However, many families of mycophagous insects such as Coleoptera [17], Diptera, and Lepidoptera also feed on multiple host species and the effects of their diet on immunity are largely unknown [18–20]. Polyphagous and mycophagous insect species also have the potential to experience different environments among fungal host species because many fungal species differ in life history, ephemerality, density, and secondary compounds [20]. We hypothesized that major effects of host species on immunity, observed in herbivorous insects, will be similar in mycophagous insects. Specifically we predicted that the variance in individual immune response would be significantly explained by adult host fungal species. If we are to draw generalizable conclusions about the role of differing host species on insect immunity, both phytophagous and mycophagous systems should be examined.

*Bolitotherus cornutus* is a mycophagous tenebrionid beetle found almost exclusively on logs infected with three species of sympatric, saprophytic fungus: *Ganoderma tsugae*, *Ganoderma applanatum*, and *Fomes fomentarius* [21, 22]. Adults feed on the surface of these fungi, females lay their eggs on the surface of the brackets, and larvae develop inside the fruiting body and emerge as adults [21]. Recent work in southern Virginia demonstrates that there is no genetic structure among patches or host fungus species, indicating natal migration among patches and host fungi may occur at a fairly high rate [23]. Adult *B*. *cornutus* are thought to spend the majority of a single breeding season on one log infected with fungus (hereafter called “patch”). Therefore, residence on a patch should be a strong predictor of current adult but not larval diet [21, 24].

The three host species may provide very different environments for adult *B*. *cornutus*. One species (*G*. *tsugae*) produces soft brackets annually which senesce and fall off the tree at the end of the breeding season while the other two species produce robust brackets that persist for years. In southwestern Virginia, the densities of *B*. *cornutus* populations among these three fungi types vary, suggesting that they may constitute differing social environments (Formica, unpublished data). These three species of fungi also cause variation in growth rates and survivorship in developing *B*. *cornutus* larvae [25], indicating that they may also differ in nutritional quality. Additionally, *B*. *cornutus* found on different species of fungi produce quantitatively different volatile secretions further suggesting that there are differences in nutrient composition and possibly chemical defenses of the host fungal species [26].

The goal of this study was to determine if individuals, sex, body size, patch, and host species predict one aspect of immune function, phenoloxidase activity, across a metapopulation of adult *B*. *cornutus*. As the three species of fungi differ in several aspects shown to affect immune function in other systems, we hypothesized that adult *B*. *cornutus* would differ the most in phenoloxidase activity among the three fungal host species.

Insects have an immune system that relies in part on the prophenoloxidase-activating system [27]. Phenoloxidase (PO) is an enzyme within a larger cascade (called the prophenoloxidase activating system) that contributes to several components of the complex immune system in insects, including melanotic encapsulation, clotting, and the production of cytotoxic chemicals, making it one of the most widely used markers in insect ecological immunity [27, 28]. The melanization response of the prophenoloxidase cascade, which results in a visible difference in color of the hemolymph, provides a way to quantify immune response in the field. Variations in phenoloxidase activity have been shown to correlate with nutrition in a variety of insect and may be driving much of the variation observed in immune differences among individuals living on different host species [9, 15, 29–31].

## Materials and Methods

Eighty-one *B*. *cornutus* were collected from 27 patches of all three fungal species (S1 Table) on Salt Pond Mountain near the Mountain Lake Biological Station in Giles County, Virginia, USA (geographic coordinates: 37.375258, -80.528747). All patches of fungi were located in two square kilometers of forest. All permits and permissions to collect *B*. *cornutus* were obtained. Patches were searched every other day during a 7 day time span (July 18–25, 2014); all animals found within this period were captured and housed individually in plastic bags with a moistened Kim Wipe® upon capture. Animals were not fed during capture to avoid altering their diet. Samples of hemolymph were collected within 24 hours of capture following the procedure described in [32]. Briefly, individuals present a tenebrio-type defensive gland from their anal sternite when exposed to human breath [33]. The gland was gently abraded with a micro-capillary tube, producing between 1–5 μL of hemolymph. This method more reliably produced hemolymph uncontaminated by other body fluids compared to hemolymph extraction via dissection. Additionally, many of these animals are part of a larger, long-term study on the heritability of social behavior therefore sacrificing individuals or obtaining more hemolymph was not feasible.

To quantify phenoloxidase activity (PO) at the field station, a filter paper immunoassay was performed [34]. Immediately after hemolymph was collected, samples were pipetted in one-microliter aliquots onto Whatman No. 2 filter paper. Hemolymph was collected from each individual only once. Depending on the amount of hemolymph collected at that time, 1–3 aliquots from the same sample were examined per individual (mean = 2.2 samples/individual). The filter paper was soaked in a solution of 10mM sodium phosphate buffer and 2mg/mL 3,4-Dihydroxy-L-phenylalanine (L-DOPA) prior to adding the hemolymph. Filter paper was kept moist for 30 minutes with the L-DOPA solution, allowed to air-dry, and then scanned on an Epson V600 flatbed scanner at 800 dpi, 48-bit color. Scanned images were converted to 8-bit gray scale and analyzed with ImageJ to determine the mean grayscale value of each hemolymph sample [35]. Because flatbed scanners record image values in a non-linear manner, we created a serial ink-dilution standard-curve. We then fit a standard curve using a third order polynomial on the log-transformed dilution values of the ink with the grayscale values taken from ImageJ (R^2^ = 0.991). To calculate an interpolated melanization index, grayscale values from the hemolymph were then interpolated using the formula of the polynomial in Graphpad Prism v6 software (San Diego, CA). After hemolymph was collected, all individuals were then photographed on the same flatbed scanner at 2400 dpi for elytra measurements. *B*. *cornutus* exhibit tonic immobility from several minutes up to a half hour when exposed to human breath; individuals were photographed while immobile by placing them dorsal side down on the scanner. Elytra lengths were then measured using ImageJ.

Although, we have previously been able to extract DNA from such secretions, we were concerned that hemolymph sampled from this region might contain defensive compounds (e.g. quinones) that could react with light and interfere with the measurement of PO activity. Therefore, we conducted a separate experiment with 30 captive beetles. Two hemolymph samples were taken from each individual. One sample was placed in a solution of buffer only (control) and the other sample was placed concurrently in the reaction described above (buffer & L-DOPA) and the results were analyzed as above. If the darkening of the samples in the PO reaction were not correlated with darkening of the same sample in the control treatment, we can conclude that our measures of PO activity from the field population were not influenced by simple darkening of defensive secretions.

To estimate the repeatability of the PO activity among individuals we used an LMM-based approach (N = 195 samples, 80 individuals) using the R package rptR in program R [36]. To determine which factors influenced immune function we constructed a general linear mixed model (lmm) in R using the lme4 package [37]. The interpolated melanization value was the dependent variable while sex, fungal species of the home patch, and elytra size (mm) were fixed effects; individual ID and patch ID were included as random effects. For the initial analysis this model was run with all two-way interactions—we excluded the three way interactions to avoid over-parameterizing our model. We then removed all of the interactions and re-ran the model to test for significance of the main effects only. In all of our lmm tests, significance for the fixed effects was determined using Wald F tests and the Kenward-Roger approximation for the denominator degrees of freedom. Wald F tests are more conservative for significance testing for fixed effects but not for random effects in linear mixed models [38]. The contribution of the random effects to the model was tested using a likelihood ratio test. Residuals of the lmm were not significantly different from normal (Shapiro-Wilk W test; W = 0.99; P = 0.129) and so no transformations were performed.

## Results and Discussion

**Fig 1:**
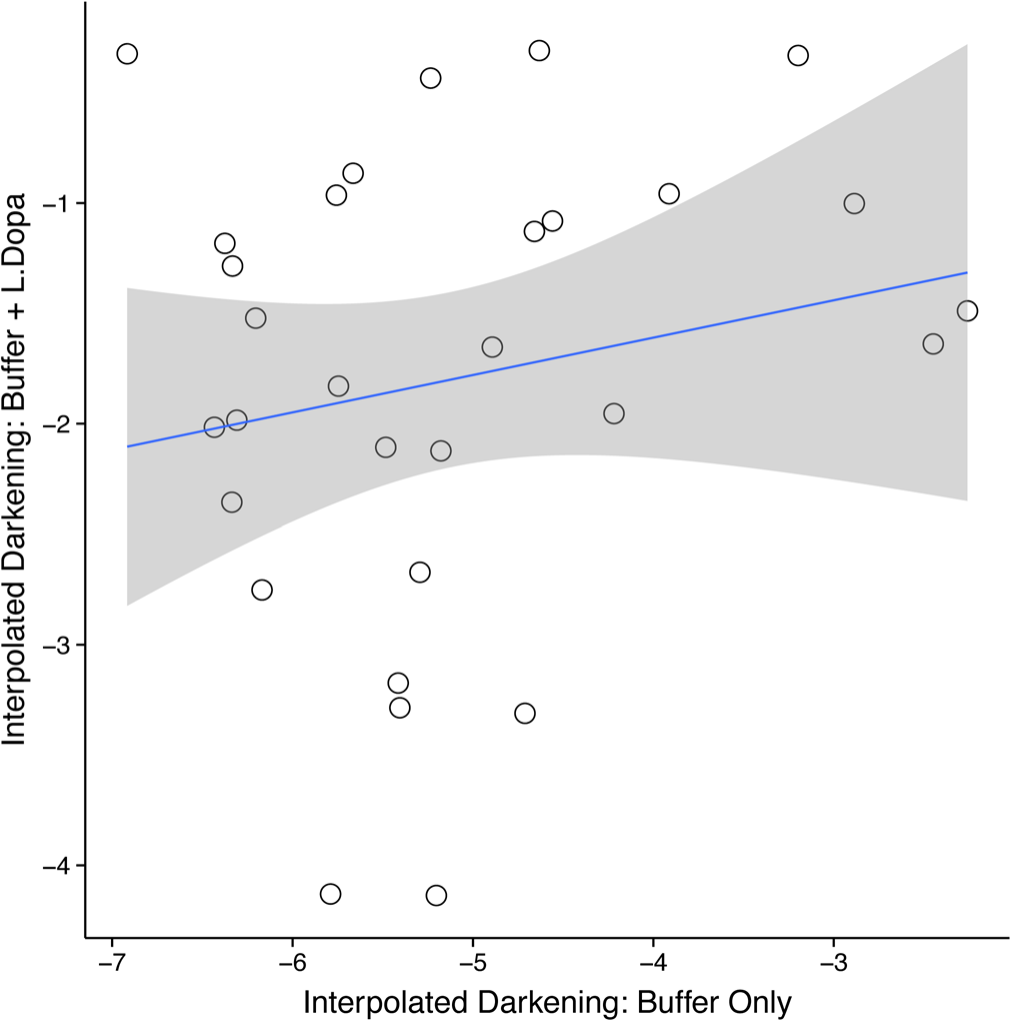
There is no correlation between the darkness of the hemolymph placed only in buffer and the darkness of hemolymph included in the immune assay (L-Dopa & Buffer; F_1, 28_ = 1.08, P = 0.31).

We found no correlation between darkness of hemolymph in buffer and darkness of hemolymph in the phenoloxidase immunoassay (F_1, 28_ = 1.08, P = 0.31; Fig 1) suggesting that the differences in darkness among samples was due to the reaction between PO activity and the L-DOPA and not simply due to darkening quinones in the defensive secretion.

**Fig 2:**
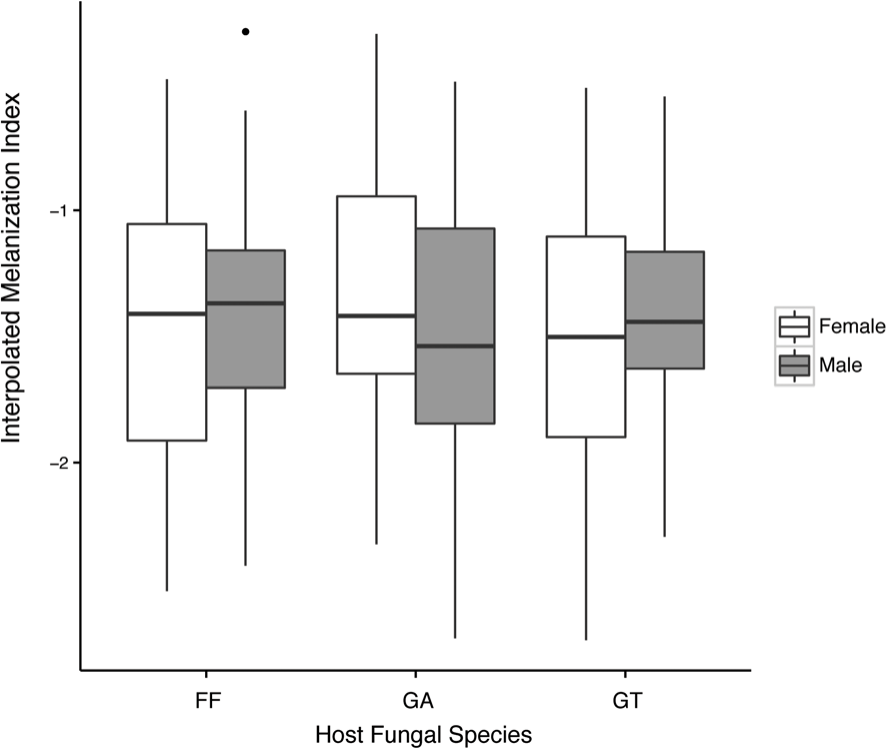
Results from the full lmm. The interpolated melanization index for each sex within the three host species (FF = *Fomes fomentarius*, GA = *Ganoderma applanatum*, GT = *Ganoderma tsugae*), demonstrating no differences among any of the categories.

Measurements of PO activity were significantly repeatable for individuals (repeatability = 0.57; 95% CI = 0.41, 0.70; P < 0.001). These results were nearly identical to the more traditional intra-class correlation estimate [39]. Not surprisingly, individual ID also explained a significant proportion of the variance of PO activity in the lmm (Table 1). This suggests there is variance in immune response within our population of *B cornutus* that might be influenced by ecological or intrinsic factors. However, the relatively low level of repeatability (0.57) suggests that our filter paper assay may have induced some within-individual variance into the assay. Surprisingly, none of the other variables—patch ID, sex, body size, host fungal species, or any of two-way interactions—significantly predicted PO activity (Table 1; Fig 2) even when the two-way interactions were removed. This result was particularly interesting since sex [40, 41], body size [42], and nutrition [30] have all been shown to cause in differences in PO activity in several insect systems.

**Table 1:** Statistical details from the general linear mixed model with interpolated melanization index as the dependent variable.

Fixed Factors	F	df	df residuals	P
Sex	0.53	1	64.0	0.47
Host species	0.14	2	68.7	0.87
Elytra length	0.26	1	66.5	0.61
Sex:Host species	0.82	2	65.0	0.44
Host species:Elytra length	0.09	2	68.5	0.91
Sex:Elytra length	0.72	1	63.6	0.40
**Random Factors**	**χ** ^**2**^	**AIC**	**df**	**P **
Individual ID	37.74	254.46	12	<0.001
Patch ID	0.00	289.21	12	1.0

Fixed factors were tested for significance using a Wald F test with Kenward-Roger df, while the random factors were tested with a likelihood ratio test. Removing the interactions had no effect on the pattern of significance for the model. Results are qualitatively similar if likelihood ratio tests were used for all significance testing.

The individual differences in PO activity we detected could be due to unmeasured factors such as age or infection status or to canalized factors influenced by natal environment or genotype (*sensu* [43]). As discussed above, host species and patch ID are representative of an individual’s current, adult environment but do not represent the historical environments experienced by these individuals. Therefore, if the natal host species were important to the development of immune function in *B*. *cornutus*, that signal would not have been detected as we only sampled adults on their current, resident host species. The majority of studies that revealed host differences in immune response examined only the effects of the environment at the larval stage [4, 12–14, 44]. Within a host species or even single fungal patch, brackets differ in size and age (and presumably nutritional quality) and so the individual differences detected may also be a result of the quality or age of brackets consumed as either an adult or a larva. However, if the nutritional quality of the natal environment was the dominant factor in determining the strength of the PO activity response, we might expect to see an effect of body size on PO activity. In holometabolous insects individuals stop growing once they eclose as adults and in other beetles adult body size has been tightly coupled with the nutritional quality of the natal environment (e.g., [45]). Alternatively, adults may move among patches more than previously thought so a single sample in the breeding season may actually reflect several adult environments over the course of an adult’s lifetime rather than the single host species upon which they were captured. Future work in this species (and other mycophagous beetles) should also focus on differences in larval immunity among host fungal species.

An alternative explanation our findings is that the fungal environments do affect adult immune response and our measure (PO activity) does not reflect the differences induced by the host species. While PO activity is a standard method to measure one aspect of immune response in insects [27, 46], it is not a measurement of total immune response and can be affected by numerous intrinsic and extrinsic factors. PO activity is part of a complex biochemical pathway and the individual variation we observed could be caused by differences in other components of the pathway and even in concentrations of inhibitors of the pathway (e.g. serpins; [27, 28, 46]). We examined only one component of the insect immune system, PO activity, and the effects of adult fungal environment on immune response might be expressed in other components of the immune system that we did not examine, such as encapsulation rate. While some studies have demonstrated correlated effects of PO activity with other immune measures, other studies have found experimentally induced changes in different components of the immune system or infection status while PO activity did not change [11, 43, 47, 48]. Therefore, we are careful not to draw conclusions about the effects of adult fungal environment on the entire immune response.

Our results do suggest, however, that the differences in PO activity driven by differences in host species found in other systems may not be generalizable across host taxa (e.g., between plants and fungus). The lack of influence of fungal host species on PO activity is somewhat surprising as others have detected effects of host species in phytophagous insects [3, 6, 9, 49]. Fungi and plants differ in number ways including physiology, structural composition, life history, and chemistry, all of which could potentially affect the relationship between host species and immune response in insects. Phytophagous insects tend to specialize on a phylogenetically restricted group of hosts while mycophagous insects tend to be broader in their host use and may have evolved differences in physiological responses to changes in host use [20, 50]. Some authors also suggest that fungal secondary compounds may not play a large role in insect host selection and so the effects of host species on insect immunity seen in phytophagous insects may not be as drastic in mycophagous insects (e.g. [51]).

It is becoming increasingly evident that host species play a large role in physiological tradeoffs that result in differences in immune function. However, these conclusions are largely being drawn from insect herbivores. In adults of the mycophagous beetle, *B*. *cornutus*, we saw no such effects. While it is impossible to draw broad comparisons from a single species in a single life stage, we conclude that mycophagous insects offer an interesting and novel system to test current hypotheses concerning host specific effects on immunity.

## Supporting Information

S1 Table Sample sizes for host species, patch, and sex used in analysis. (DOCX)

S2 Table Raw data (.csv) table used in analysis. (CSV)
