# Supplementary material for: No Effect of Host Species on Phenoloxidase Activity in a Mycophagous Beetle
Source: PLoS One. 2015 Oct 29;10(10):e0141167. doi: 10.1371/journal.pone.0141167 (PMC4625955; doi:10.1371/journal.pone.0141167)
Supplement: S1 Table — (DOCX) [file pone.0141167.s001.docx]

| Host Fungal Species | Patch ID | Number of Females | Number of Males | Total |
| --- | --- | --- | --- | --- |
| *Fomes fomentarius* |  |  |  |  |
|  | PER-189 | 4 | 2 |  |
|  | PER-221 | 1 | 1 |  |
|  | PER-227 | 1 | 1 |  |
|  | PER-228 | 0 | 1 |  |
|  | PER-235 | 0 | 1 |  |
|  | PER-295 | 0 | 1 |  |
|  | PER-299 | 1 | 0 |  |
|  | PER-930 | 0 | 2 |  |
| Totals | 8 patches | 7 | 9 | 16 |
| *Ganoderma applanatum* |  |  |  |  |
|  | BTP-1 | 6 | 3 |  |
|  | PER-190 | 5 | 2 |  |
|  | PER-231 | 1 | 0 |  |
|  | PER-242 | 4 | 7 |  |
|  | PER-912 | 1 | 1 |  |
|  | PER-931 | 2 | 1 |  |
|  | PER-944 | 0 | 1 |  |
| Totals | 7 patches | 19 | 15 | 34 |
| *Ganoderma tsugae* |  |  |  |  |
|  | BTP-2 | 1 | 2 |  |
|  | CLAY-01 | 4 | 0 |  |
|  | DOL-04 | 0 | 1 |  |
|  | DOL-06 | 1 | 0 |  |
|  | DOL-09 | 1 | 0 |  |
|  | DOL-10 | 2 | 0 |  |
|  | DOL-18 | 2 | 0 |  |
|  | PER-240 | 0 | 1 |  |
|  | PER-260 | 1 | 0 |  |
|  | PER-264 | 2 | 2 |  |
|  | PER-929 | 1 | 0 |  |
|  | PER-949 | 4 | 6 |  |
| Totals | 12 patches | 19 | 12 | 31 |
| Sum totals  for analysis | 27 Patches | 45 | 36 | 81 |

Supp. Table 1. Sample sizes for host species, patch, and sex used in analysis.
